# PZT-Film-Based Piezoelectric Micromachined Ultrasonic Transducer with I-Shaped Composite Diaphragm

**DOI:** 10.3390/mi13101597

**Published:** 2022-09-26

**Authors:** Qing Yu, Guoxiang Fan, Wei Ren, Qingqing Fan, Jinming Ti, Junhong Li, Chenghao Wang

**Affiliations:** 1 State Key Laboratory of Acoustics, Institute of Acoustics, Chinese Academy of Sciences, Beijing 100190, China; 2 University of Chinese Academy of Sciences, Beijing 100049, China

**Keywords:** pMUT, I-shaped composite diaphragm, loop gain (loss), resonant frequency

## Abstract

We proposed a PZT-film-based piezoelectric micromachined ultrasonic transducer (pMUT) with an I-shaped composite diaphragm to improve the sensitivity and resonant frequency of pMUTs with the same diaphragm area. The finite element method (FEM) simulation results indicated that the pMUT with an I-shaped composite diaphragm had relatively high sensitivity and resonant frequency. The pMUT with an I-shaped diaphragm had a 36.07% higher resonant frequency than a pMUT with a circular diaphragm. The pMUT with an I-shaped diaphragm had a 3.65 dB higher loop gain (loss) than a pMUT with a rectangular diaphragm. The piezoelectric layer thickness of the pMUT with an I-shaped composite diaphragm was then optimized. Maximum loop gain (loss) was reached when the piezoelectric layer thickness was 8 μm. The pMUT with an I-shaped composite diaphragm was fabricated using the MEMS method, and its performance was evaluated.

## 1. Introduction

Micromachined ultrasonic transducers (MUTs) have important applications in medical ultrasonic arrays, gesture recognition, endoscopic imaging, and fingerprint recognition, among other fields [1,2,3,4]. Compared with traditional ultrasonic transducers, MUTs have a small size, light weight, high integration, low cost, and other merits. They have become one of the present hotspots in the ultrasonic transducer field. MUTs are currently categorized primarily as pMUT [5,6,7] and capacitive micromachined ultrasonic transducer (cMUT) [8,9,10,11].

A pMUT is driven by a piezoelectric layer and transmits and receives ultrasonic waves via the bending vibration of the diaphragm. A cMUT is a capacitive structure consisting of a diaphragm and back-plate. Through the vibration of the membrane, the capacitance between the diaphragm and back-plate is changed to transmitting and receiving sound waves. According to the capacitance impedance formula Z = 1/jwC, when the capacitance becomes larger, the internal resistance will decrease. On the basis of the working principle of a cMUT, its capacitive gap medium is close to vacuum, so ε_cPMUT_ = ε_0_ = 8.85 × 10^−12^ (F/M). However, the relative permittivity of PZT-5H is 1700. Therefore, the capacity value of a pMUT is 1700 times that of a cMUT. This means that a pMUT has a higher capacitance and lower impedance to better match the circuit. In addition, compared to a cMUT, a pMUT has the advantages of no bias voltage, simple electrical impedance matching, dust-proofing, and water resistance, thus attracting a great deal of academic interest [12,13,14]. pMUTs have a diverse range of potential applications in medical ultrasound and other fields [15,16,17]. Important pMUT performance indicators include the imaging resolution and sensitivity, and the resonant frequency is directly related to the imaging resolution. The higher the resonant frequency, the higher the imaging resolution. High sensitivity enables better image clarity and contrast.

Resonant frequency and sensitivity are two important indicators of pMUTs. Normally, the resonant frequency is increased by changing the size or thickness of the pMUT, but this will lead to the reduction of sensitivity. Haoran Wang et al. designed seven pMUT elements with different resonant frequencies by changing the radius of the vibration membrane [18]. When the resonant frequency increases from 1.15 MHz to 7.80 MHz, the sensitivity decreases from 130 nm/V to 8 nm/V. Similarly, in our previous study [19], the structure optimization of the square diaphragm improved the sensitivity of the pMUT but reduced the resonant frequency simultaneously.

However, by etching holes or slots in the membrane, the residual stress of the diaphragm is reduced, and the vibration amplitude is increased. Compared with the traditional pMUT with a similar shape, it can improve the resonant frequency and sensitivity concurrently. Tao Wang et al. etched circular holes on the square diaphragm [20]. The output sound pressure of the proposed pMUT could be increased by about 5.3 dB and the resonant frequency could be increased by 2%. Xuying Chen et al. proposed a pMUT structure with V-shaped springs [21]. With the design of a V-shaped hole, the output sound pressure of a pMUT can be increased by about 23 dB, and the resonant frequency is increased at the same time. However, etching holes or slots in the membrane greatly increases the fabrication process complexity.

According to the above problems, we proposed a PZT-film-based pMUT with an I-shaped composite diaphragm. The I-shaped diaphragm structure is formed by etching part of the piezoelectric layer that is not covered by electrodes. The effective bending of the piezoelectric layer is enhanced, and the residual stress generated during PZT deposition is partly eliminated. Thus, the pMUT can obtain high resonant frequency while the sensitivity is essentially unchanged. The fabrication process complexity is greatly reduced because there are no holes or slots in the membrane. The loop gain (loss) can comprehensively reflect the transmitting and receiving sensitivity of the pMUT and is obtained by adding the transmitter voltage response and the receiving sensitivity. MUTs have a smaller radiation area than conventional ultrasonic transducers, so their loop gain (loss) is more dependent on the transmitting sensitivity of the pMUT. PZT is a common piezoelectric material for pMUTs with a high piezoelectric coefficient d_31_, which is conducive to enhanced transmitting sensitivity. Therefore, we chose PZT material as the piezoelectric layer of the pMUT. The FEM was used to compare and analyze composite diaphragms of square, rectangular, circular, and I-shape. The thickness of the piezoelectric layer of the pMUT with an I-shaped diaphragm was optimized. Based on the process preparation study, the pMUT was designed and fabricated, and its transmitter voltage response and receiving sensitivity were evaluated.

## 2. Design of Transducer

### 2.1. Structure and Parameters

A pMUT is composed of a piezoelectric layer (PZT), a top electrode (Au/Cr), a bottom electrode (Pt/Ti), a silicon layer, a silicon oxide layer, and substrate. It transmits and receives ultrasonic waves through the diaphragm’s bending vibration. During simulation, the top and bottom electrode layers’ effect on vibration was ignored. The structure of the piezoelectric composite diaphragm consists of a silicon layer, a silicon oxide layer, and a piezoelectric layer (PZT).

**Figure 1:**
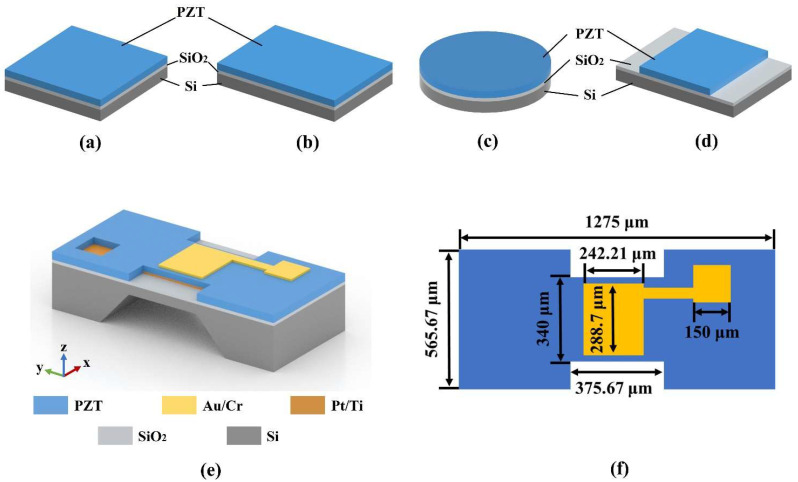
Schematic structure of pMUT’s composite diaphragm with (**a**) square; (**b**) rectangular; (**d**) circular; (**c**) I-shape. (**e**) Schematic structure of the pMUT with I-shaped composite diaphragm. (**f**) Structural parameters of the I-shaped diaphragm.

The square and circular composite diaphragm are the common diaphragm structures of pMUTs [22,23]. To obtain a high resonant frequency and sensitivity with the same diaphragm area, we investigated a pMUT with a square (Figure 1a), rectangular (Figure 1b), and circular (Figure 1c) composite diaphragm and proposed an I-shaped composite diaphragm (Figure 1d). Figure 1e,f depicts the proposed PZT-based pMUT with an I-shaped composite diaphragm. A portion of the diaphragm’s piezoelectric layer was etched to form an I-shaped PZT film to obtain a high resonant frequency and sensitivity.

To investigate the effects of various diaphragm structures on the resonant frequency and sensitivity of the pMUT, the modals and simulated analyses of square, rectangular, circular, and I-shaped diaphragm structures with the same area were implemented by means of FEM. Geometric models of four distinct composite diaphragm structures of a pMUT were built. The structure parameters and material characteristics used in the FEM simulation are listed in Table 1 and Table 2.

The developed pMUT is mainly used in endoscopic ultrasound medical imaging and portable medical imaging. It works in a liquid environment. Considering the short circuit problem in the water, the test environment with absolute ethanol was chosen. Therefore, the absolute ethanol domain was also selected in the simulation analysis. A perfectly matched layer (PML) was established around the absolute ethanol domain (1168 m/s in sound velocity). The radius of the absolute ethanol domain was 1000 μm, and the thickness of the PML was 100 μm. Loading and boundary conditions were added to the model. A fixed boundary condition was added to the structure’s diaphragm. For simulation of the transmitter voltage response, a voltage of 10 V was applied to the upper surface of the PZT piezoelectric layer of the pMUT, and the lower surface was grounded. For the simulation of the receiving sensitivity, we applied a pressure of 1 Pa on the diaphragm. The outer surface of the absolute ethanol domain was set as the far-field boundary. The far-field calculation determined the distance between the sound pressure and the transducer to be 20 mm.

### 2.2. Impact of Membrane and PZT Shape on Resonant Frequency

The FEM modal analysis was performed on the composite diaphragm structure. The resonance frequencies of the pMUT with four composite diaphragms in the air, DI water, and absolute ethanol domains are shown in Table 3. Due to the effect of the fluid medium’s mass, the resonant frequency of the device in the DI water and absolute ethanol domains were significantly lower than in the air vibration environment. Moreover, the resonant frequency of the circular diaphragm decreased the most, while the I-shaped diaphragm decreased the least.

Xiaoyue Jiang et al. studied the resonant frequency of the pMUT with a square and circular composite diaphragm in a fluid medium [24].

The mass per unit area *μ* of the pMUT with a square and circular diaphragm is:

(1) $$\mu =\sum_{n=1}^{3}t_{n}\rho_{n}$$

where *t_n_* is the thickness of the *n*th layer and $\rho_{n}$ is the density of the *n*th layer.

Therefore, the resonant frequency of the pMUT with a square or circular diaphragm in the fluid medium is:

(2) $$f_{0,fluid}\approx \frac{f_{0,air/vacuum}}{\sqrt{1+0.34\frac{\rho_{fluid}a}{\mu }}}$$

where $\rho_{fluid}$ is the density of the fluid medium; *a* is the side length or diameter.

According to Formula (2), the greatest decrease in the resonant frequency of a circular diaphragm is due to its larger diameter in the same area of the membrane. While the reason why the resonance frequency of the I-shaped diaphragm decreases less may be that the effective size of the diaphragm decreases due to the partial etching of the PZT film.

### 2.3. Electrode Optimization

When external acoustic pressure is applied to any of the four types of diaphragms described previously, the stresses in the diaphragm change gradually from edge to center. The stresses in the central and edge areas are opposite. When the electrodes cover the entire piezoelectric layer, positive and negative stresses cause the opposite charge, which is offset, thereby decreasing the pMUT’s receiving sensitivity. Therefore, the electrode distribution of the pMUTs was optimized by analyzing the stress distribution in the mid-plane of the PZT layer.

**Figure 2:**
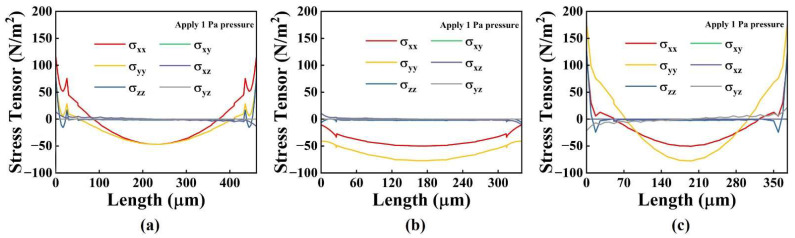
The stress distribution of (**a**) square diaphragm; (**b**) length direction of I-shaped diaphragm; (**c**) width direction of I-shaped diaphragm.

Figure 2 depicts the FEM simulation analysis of the stress distribution of the PZT piezoelectric layer in the different structures when 1 Pa of pressure is applied to the diaphragm.

**Figure 3:**
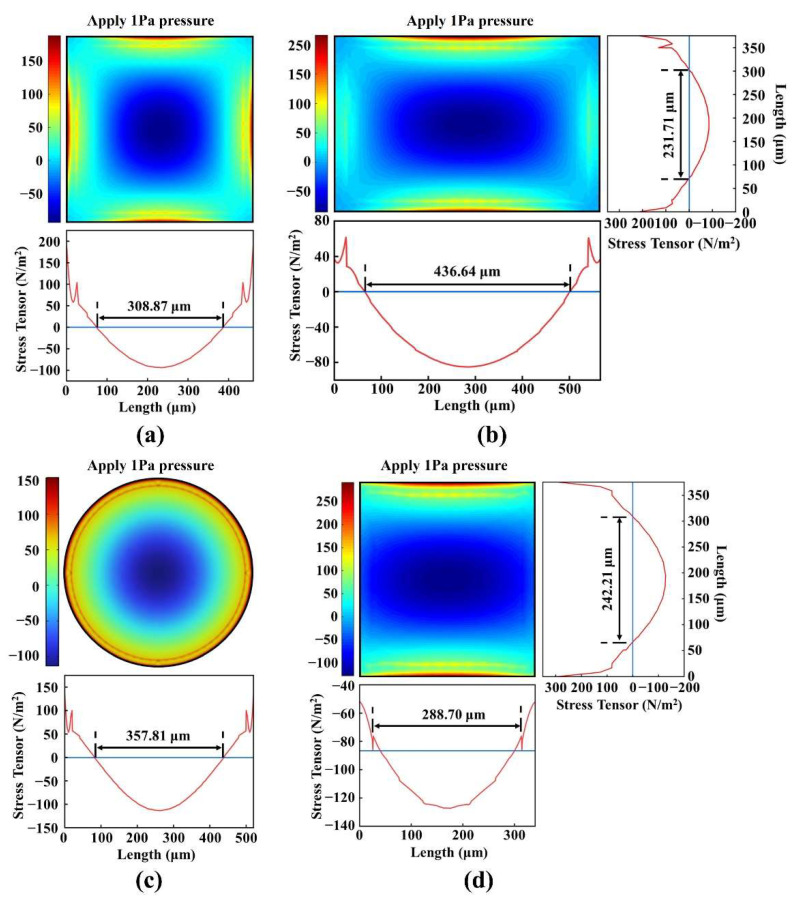
The sum stress distribution of σ_xx_ and σ_yy_ in the PZT piezoelectric layer of (**a**) square diaphragm; (**b**) rectangular diaphragm; (**c**) circular diaphragm; (**d**) I-shaped diaphragm.

The results indicated that the σ_xx_ and σ_yy_ stress components were significantly larger than those in the other directions, so we neglected the stress components in the other directions during the simulation. Figure 3 shows the sum stress distribution of σ_xx_ and σ_yy_ in the PZT piezoelectric layer for the four composite diaphragm structures. The square, rectangular, and circular PZT films had positive stress in the edge area and negative stress in the center area, which is consistent with the width direction of the I-shaped PZT film. We took the position where the stress component in the length direction of the I-shaped PZT film decreased sharply as the electrode length distribution. Therefore, the electrode area was 308.87 × 308.87 μm^2^ for the square diaphragm; 436.64 × 231.71 μm^2^ for the rectangular diaphragm; π × (178.9)^2^ μm^2^ for the circular diaphragm; and 288.70 × 242.21 μm^2^ for the I-shape diaphragm. Based on the results of optimizing the electrodes, the maximum electrode area was 10.12 × 10^−^^4^ μm^2^, and the minimum electrode area was 6.99 × 10^−^^4^ μm^2^. The difference between them was 1.45 times. The corresponding capacitance values were also only 1.45 times different. Therefore, the design of these four different structures had little influence on the electrical impedance matching of the pMUT.

### 2.4. Impact of Diaphragm Structure on pMUT Performance

The transmitter voltage response and receiving sensitivity were analyzed for square, rectangular, circular, and I-shaped diaphragms with the same area based on the electrode optimization. Table 4 shows the resonant frequency, transmitter voltage response (S_VL_), receiving sensitivity (M_el_), and loop gain (loss) (G) of pMUTs with four distinct diaphragms. The impact of these four distinct diaphragm structures on the performance of the pMUT was investigated.

The high resonant frequency and high sensitivity could not be obtained simultaneously under the same diaphragm area. A pMUT tends to decrease its resonant frequency while increasing its transmitting and receiving sensitivity. According to Table 4, the I-shaped diaphragm had the highest resonant frequency, while the circular diaphragm had the lowest.

The I-shaped design of the diaphragm increased its bending stiffness. Consequently, its resonant frequency was 15.28%, 1.22%, and 36.07% greater than those of the square, rectangular, and circular structures, respectively. By removing the PZT piezoelectric layer without an electrode, the effective bending of the PZT diaphragm was enhanced, and the residual stress generated during the deposition of the PZT films was relieved. As a result, the I-shaped diaphragm had the highest transmitter voltage response, which was 166.91dB. I-shaped diaphragm loop gain (loss) was nearly identical to that of the square (only 0.2 dB difference) and circular (only 1.57 dB difference), which was greater than that of the rectangular (3.65 dB). It showed that the design of the I-shaped diaphragm can improve the resonant frequency and ensure high sensitivity.

## 3. Microfabrication of pMUT

We prepared PZT piezoelectric layers using the 0–3 method. PZT films are prepared by combining composite sol-gel. The PZT slurry is mixed by PZT powder and sol. The addition of powder can reduce the stress in PZT film, so as to obtain a relatively thick PZT film. The detailed preparation process is described in our other article [25].

**Figure 4:**
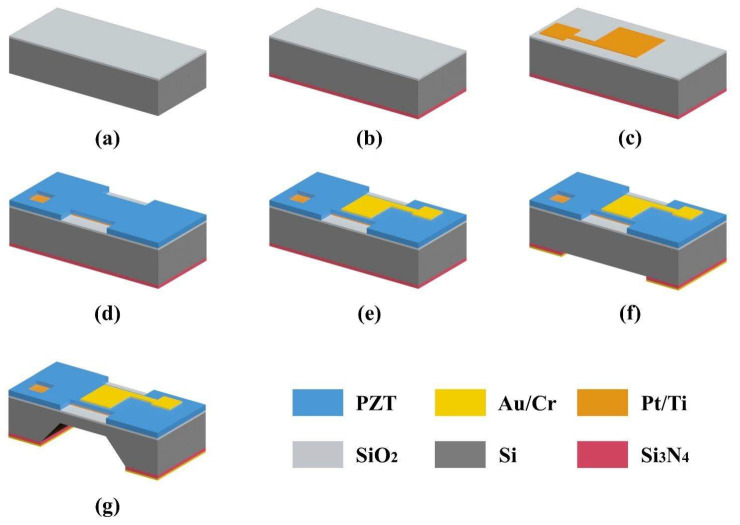
Fabrication process of the pMUT.

As shown in Figure 4, the pMUT with an I-shaped diaphragm was fabricated using the MEMS method. The (100) silicon substrate was coated with 0.2-μm-thick silicon oxide using thermal oxidation. As shown in Figure 4a, the backside silicon oxide was then etched. As shown in Figure 4b, a 0.6-μm-thick silicon nitride (Si_3_N_4_) layer was then deposited on the backside as part of the bulk silicon etching mask layer. The Pt/Ti bottom electrode was then deposited and patterned on the silicon oxide layer, as shown in Figure 4c. Next, a PZT piezoelectric layer was prepared using the 0–3 method and patterned as shown in Figure 4d. The top electrode Au/Cr was then deposited on the PZT layer and patterned, as shown in Figure 4e. As depicted in Figure 4f, after deposition of the Au/Cr films, an Au/Cr/Si_3_N_4_ composite mask for bulk silicon etching was fabricated by successively etching Au/Cr and Si_3_N_4_ films. As shown in Figure 4g, the silicon substrate was etched by wet bulk silicon to form a silicon diaphragm by controlling etching time. Finally, the device preparation has been completed.

## 4. Results and Discussion

### 4.1. Finite Element Analysis

To further optimize the performance of the pMUT with an I-shaped diaphragm structure, we analyzed the impact of various piezoelectric layer thicknesses on the pMUT’s sensitivity. In the FEM modeling procedure, the thickness of the PZT piezoelectric layer was scanned at 3 μm intervals, and values ranged from 5 to 20 μm.

**Figure 5:**
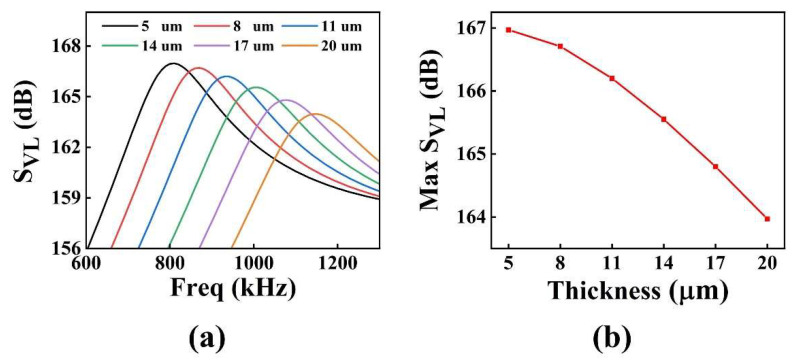
The pMUT with I-shaped diaphragm: (**a**) transmitter voltage response curve graph; (**b**) variation of transmitter voltage response at resonant frequency with piezoelectric layer thickness.

Figure 5 depicts (a) the transmitter voltage response and (b) the variation of the transmitter voltage response at resonant frequency with different piezoelectric layer thickness for the pMUT with an I-shaped diaphragm. As the thickness of the PZT piezoelectric layer increased, the bending stiffness of the diaphragm also increased, resulting in a gradual increase in the resonant frequency of the pMUT. At the same time, the effective bending decreased, resulting in a decreasing trend in the transmitter voltage response.

**Figure 6:**
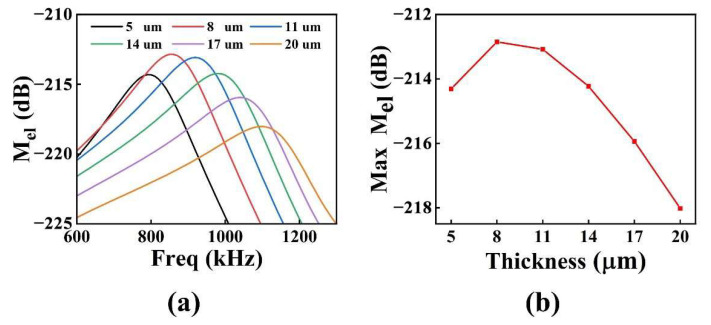
The pMUT with I-shaped diaphragm: (**a**) receiving sensitivity curve graph; (**b**) receiving sensitivity at resonant frequency variation with piezoelectric layer thickness.

Figure 6 shows graphs of (a) receiving sensitivity and (b) receiving sensitivity at resonant frequency variations with different piezoelectric layer thickness for the pMUT with an I-shaped diaphragm. With an increase in the thickness of the PZT piezoelectric layer, the receiving sensitivity at resonant frequency first increased and then decreased. When the thickness of the piezoelectric layer was 8 μm, the maximum receiving sensitivity of pMUT was −212.85 dB. The receiving sensitivity is dependent on the capacitance of the pMUT, the stress level of the piezoelectric layer, and the neutral surface position of the piezoelectric composite diaphragm; therefore, there is an optimal thickness value to maximize the receiving sensitivity.

**Figure 7:**
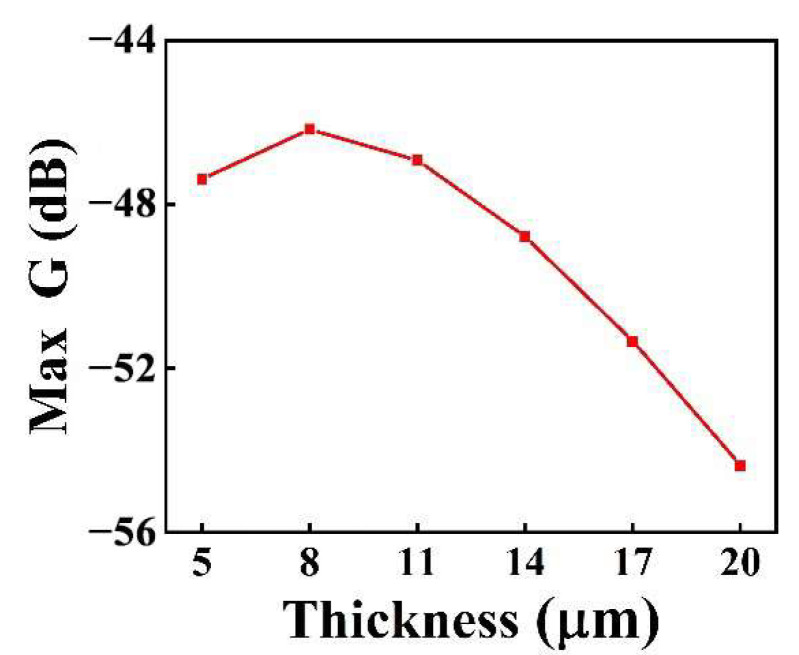
The loop gain (loss) at resonant frequency of the I-shaped diaphragm pMUT varies with the thickness of the piezoelectric layer.

Figure 7 shows the variation of the loop gain (loss) at resonant frequency with the thickness of the piezoelectric layer for the I-shaped diaphragm pMUT. As the thickness of the PZT piezoelectric layer increased, the loop gain (loss) initially increased, then decreased. The maximum loop gain (loss) of the pMUT was −46.17 dB when the piezoelectric layer PZT had a thickness of 8 μm, which corresponds to the highest receiving sensitivity.

### 4.2. Results of the pMUT Experiment

According to the simulation result, when the thickness of the PZT piezoelectric layer was 8 μm, the loop gain (loss) of I-shaped PMUT was the highest. However, the thicker piezoelectric layer needed a long chemical etching time in the patterning process, which affected other parts of the device. Therefore, considering the compatibility of the pattern of the PZT piezoelectric layer with the MEMS process, the thickness of the PZT layer was selected as 4 μm.

After the pMUT with I-shaped diaphragm was prepared, the die was cut and glued onto a printed circuit board (PCB), electrically connected via ultrasonic pressure welding, and encapsulated in a metal box to achieve electromagnetic shielding. The composite diaphragm of the pMUT was exposed by the window of the metal box.

Sensitivity test experiments were conducted on the developed pMUT, and the transducer transmitting and receiving test experiments were carried out in absolute ethanol solution.

In the transmitting sensitivity test, the developed pMUT served as the transmitting transducer, while the calibrated hydrophone served as the receiving transducer. The excitation signal consisted of 10 sine waves at 850 kHz with a peak-to-peak voltage of 10 V.

**Figure 8:**
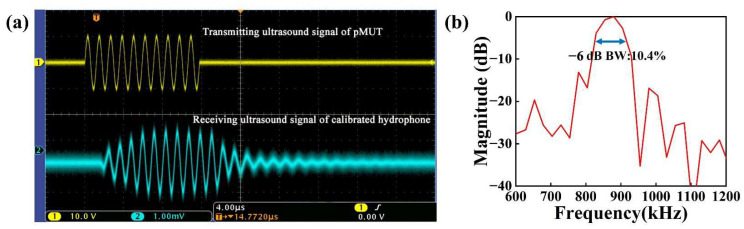
(**a**) Measurement results of transmitting sensitivity of a pMUT with an I-shaped diaphragm; (**b**) the frequency response of a pMUT with I-shaped diaphragm.

The waveform of the signal received by the developed pMUT is shown in Figure 8a (the *x*-axis scale stands for 4.00 μs; the *y*-axis scale of the transmitting ultrasound signal and receiving ultrasonic signal stands for 10.0 V and 1.00 mV, respectively). After calibration, the transmitting sensitivity of the pMUT was 2.1812 Pa/V, i.e., 126.77 dB (ref. 1 μPa/V) at 20 mm equivalent distance. As shown in Figure 8b, the calibrated hydrophone had a −6 dB bandwidth of 10.4% with a center frequency of 870.52 kHz.

In the receiving sensitivity test, the excitation signal was a 10-cycle sine wave at 850 kHz with a peak-to-peak value of 10 V generated by the signal generator. The received signal was acquired directly by an oscilloscope.

**Figure 9:**
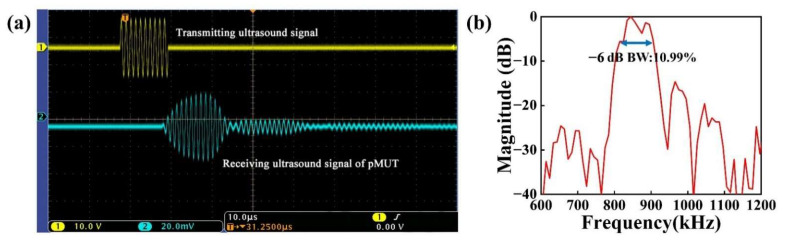
(**a**) Measurement results of receiving sensitivity of a pMUT with an I-shaped diaphragm; (**b**) the frequency response of a pMUT with I-shaped diaphragm.

**Figure 10:**
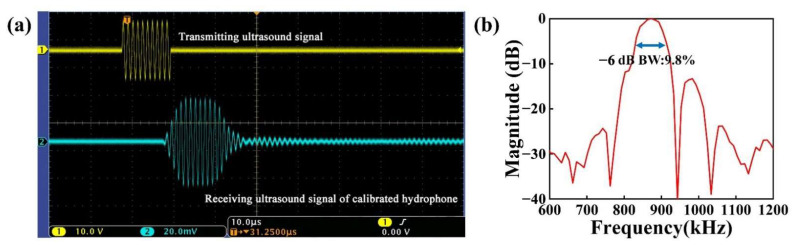
(**a**) Measurement results of receiving sensitivity of a calibrated hydrophone; (**b**) the frequency response of a calibrated hydrophone.

Figure 9a and Figure 10a depict the received signal of the developed pMUT and the received signal of the calibrated hydrophone (the *x*-axis scale stands for 10.0 μs; the *y*-axis scale of the transmitting ultrasound signal and receiving ultrasonic signal stands for 10.0 V and 20.0 mV, respectively). The designed pMUT’s received signal amplitude was 25.6 mV. The received signal amplitude of the calibrated hydrophone was 32.2 mV. The calculation result showed that the receiving sensitivity of the device was 0.47 μV/Pa, i.e., −246.56 dB (ref. 1 V/μPa). As shown in Figure 9b and Figure 10b, the bandwidth of the designed pMUT was 10.99%, with a center frequency of 860.26 kHz. The calibrated hydrophone had a −6 dB bandwidth of 9.8% with a center frequency of 872.62 kHz.

## 5. Conclusions

We proposed a PZT-film-based pMUT with an I-shaped composite diaphragm to improve the sensitivity and resonance frequency (high imaging resolution) simultaneously. The pMUT with four different diaphragms was analyzed by the FEM. I-shaped diaphragms had the highest resonant frequency, which was 15.28%, 1.22%, and 36.07% higher than those of square, rectangular, and circular, respectively. The I-shaped diaphragm loop gain (loss) was similar to that of the square and circular structure and 3.65 dB higher than that of the rectangular structure. Under the same diaphragm area, the I-shaped diaphragm could simultaneously achieve a higher resonant frequency and higher sensitivity. The performance of the I-shaped diaphragm with different PZT piezoelectric layer thicknesses was then optimized and enhanced. With an increase in the PZT thickness of the piezoelectric layer, the transmitter voltage response of the pMUT exhibited a general downward trend, while the receiving sensitivity exhibited an initial increase followed by a decrease. The loop gain (loss) reached its maximum when the piezoelectric layer PZT had a thickness of 8 μm. Considering the compatibility of the pattern of the PZT layer with the MEMS process, the PZT piezoelectric layer thickness of the device was selected as 4 μm. In absolute ethanol solution, the transmitting sensitivity and receiving sensitivity of the pMUT with an I-shaped diaphragm were measured. The results indicated that the transmitting sensitivity of the pMUT based on a PZT piezoelectric layer with an I-shaped diaphragm was 126.77 dB (ref. 1 μPa/V), while the receiving sensitivity was -246.56 dB (ref. 1 V/μPa). In future work, a pMUT with a PZT piezoelectric layer thickness of 8 μm will be prepared after improving the compatibility of the pattern of the PZT layer with the MEMS process, so as to improve the performance of the pMUT.

## Figures and Tables

**Table 1:** Structure parameters of four types of the composite diaphragm.

Diaphragm Structure	Length of Diaphragm (μm)	Width of Diaphragm (μm)	Length of PZT (μm)	Width of PZT (μm)
Square	461.00	461.00	461.00	461.00
Rectangular	565.67	375.67	565.67	375.67
I-shape	565.67	375.67	340.00	375.67
**Diaphragm Structure**	**Radius of diaphragm** **(μm)**		**Radius of PZT** **(μm)**	
Circular	260.08		260.08	

**Table 2:** Material characteristics and thickness used in FEM simulation.

Material	Property	Value	Units
Si	Thickness	18	μm
	Young’s modulus	167	GPa
	Density	2.33	10^3^ kg/m^3^
	Poisson’s ratio	0.28	/
SiO_2_	Thickness	0.2	μm
	Young’s modulus	72	GPa
	Density	2.30	10^3^ kg/m^3^
	Poisson’s ratio	0.16	/
PZT	Thickness	6	μm
	Density	7.50	10^3^ kg/m^3^
	Elasticity matrix	$\left[\begin{array}{cccccc} 1.27 & 0.80 & 0.85 & 0 & 0 & 0\\ 0.80 & 1.27 & 0.85 & 0 & 0 & 0\\ 0.85 & 0.85 & 1.17 & 0 & 0 & 0\\ 0 & 0 & 0 & 0.23 & 0 & 0\\ 0 & 0 & 0 & 0 & 0.23 & 0\\ 0 & 0 & 0 & 0 & 0 & 0.23 \end{array}\right]$	10^3^ GPa
	Coupling matrix	$\left[\begin{array}{cccccc} 0 & 0 & 0 & 0 & 17.03 & 0\\ 0 & 0 & 0 & 17.03 & 0 & 0\\ -6.62 & -6.62 & 23.24 & 0 & 0 & 0 \end{array}\right]$	C/m^2^
	Relative dielectric constant	$\left[\begin{array}{ccc} 1704.4 & 0 & 0\\ 0 & 1704.4 & 0\\ 0 & 0 & 1433.6 \end{array}\right]$	/

**Table 3:** The resonant frequencies of the pMUT with four composite diaphragms in the air, DI water, and absolute ethanol domains.

Diaphragm Structure	Air Domain (kHz)	DI Water Domain (kHz)	Absolute Ethanol Domain (kHz)
Square	1074	660	720
Rectangular	1203	750	820
Circular	963	570	610
I-shape	1182	760	830

**Table 4:** The performances of four pMUTs with different diaphragms in the absolute ethanol domain.

Diaphragm Structure	Resonant Frequency (kHz)	Transmitter Voltage Response (dB)	Receiving Sensitivity (dB)	Loop Gain (Loss) (dB)
Square	720	166.73	−213.13	−46.41
Rectangular	820	166.43	−216.46	−50.25
Circular	610	166.55	−211.33	−45.03
I-shape	830	166.91	−213.52	−46.60

## Data Availability

Not applicable.
